# Time taken to resume activities of daily living after transsphenoidal surgery for pituitary tumors

**DOI:** 10.1038/s41598-023-31203-9

**Published:** 2023-03-13

**Authors:** Jeong-A. Lee, Eun-Young Tak, Hyang Lan Lim, Seonghee Oh, Hyojung Sim, Hye-Ok Choi, Doo-Sik Kong

**Affiliations:** 1 Department of Nursing, Samsung Medical Center, Seoul, Korea; 2 Department of Neurosurgery, Samsung Medical Center, Sungkyunkwan University School of Medicine, 81 Irwon-ro, Gangnam-gu, Seoul, 06351 Korea

**Keywords:** Health care, Neurology

## Abstract

Postoperative management after transsphenoidal surgery (TSS) is important; however, the guidelines for resuming daily activities after TSS are insufficient. This study aimed to examine the time to return to activities of daily living (ADL) after TSS for pituitary tumors. A 4-month prospective data collection was completed for 114 of 117 patients who underwent TSS for pituitary tumors from April to July 2021. The time when the patient returned to ADL after surgery was measured using the self-recording sheet. More than 97% and 92% of the patients returned within 1 month (median: within 7 days) for the elements of basic ADL and within 2 months (median: within 15 days) for the elements of instrumental ADL, excluding a few. Notably, 73.3% of patients returned to work within 4 months. The median time for the activities included 64 days for washing hair head down, 44 days for blowing nose, 59 days for lifting heavy objects, and 102 days for sexual activity. For patients who received extended-TSS or had postoperative problems, the time to return was delayed. Based on these results, it will be possible to provide practical information and guidelines on the time to return to ADL after TSS in pituitary tumor patients.

## Introduction

Transsphenoidal surgery (TSS) has remained the standard surgical treatment for pituitary adenoma (PA) since its introduction in the early 1900s, and its indications have been expanded to include lesions other than PA^1^. Although the outcomes of this surgical approach have improved over time, neurosurgical complications such as postoperative cerebrospinal fluid (CSF) leaks or meningitis and endocrine dysfunction such as diabetes insipidus (DI) should be monitored^1,2^. The common reasons for 30-day readmission after TSS were epistaxis, hyponatremia, CSF leak, and other medical conditions, which added significant costs to the treatment^3^. Proper postoperative care and patient education are important to prevent and minimize the complications associated with TSS^1–3^.

Several patients inquire whether they can return to their daily lives after surgery, or when their function will return to the pre-operative levels. Patients anticipated a cure after TSS and were satisfied with the surgical results. Several patients reported receiving adequate perioperative information; however, half of them felt that they had not received adequate postoperative information. Some aspects of post-TSS care still need improvement^4^.

Based on previous studies on the time to return to activities of daily living (ADL), a consensus was reached among pituitary surgeons on the recommendations for the time to return to specific activities after TSS^5^. Other than that, the return-to-work rates were measured, and a few behavior guidelines were introduced as part of the recovery protocol after TSS^6–8^. Research on the time to return to comprehensive and specific ADL after TSS is insufficient. It is necessary to prepare a basis for reducing the postoperative complications after TSS, resolving uncertainty about the postoperative recovery process, and helping them to return to their specific, practical, and comprehensive ADL in the safest and fastest time after TSS. Therefore, this study aimed to present the basic data needed to provide information and guidelines for the appropriate return time by examining the time to return to ADL after TSS in patients with pituitary tumors.

## Results

### General and clinical characteristics

**Figure 1:**
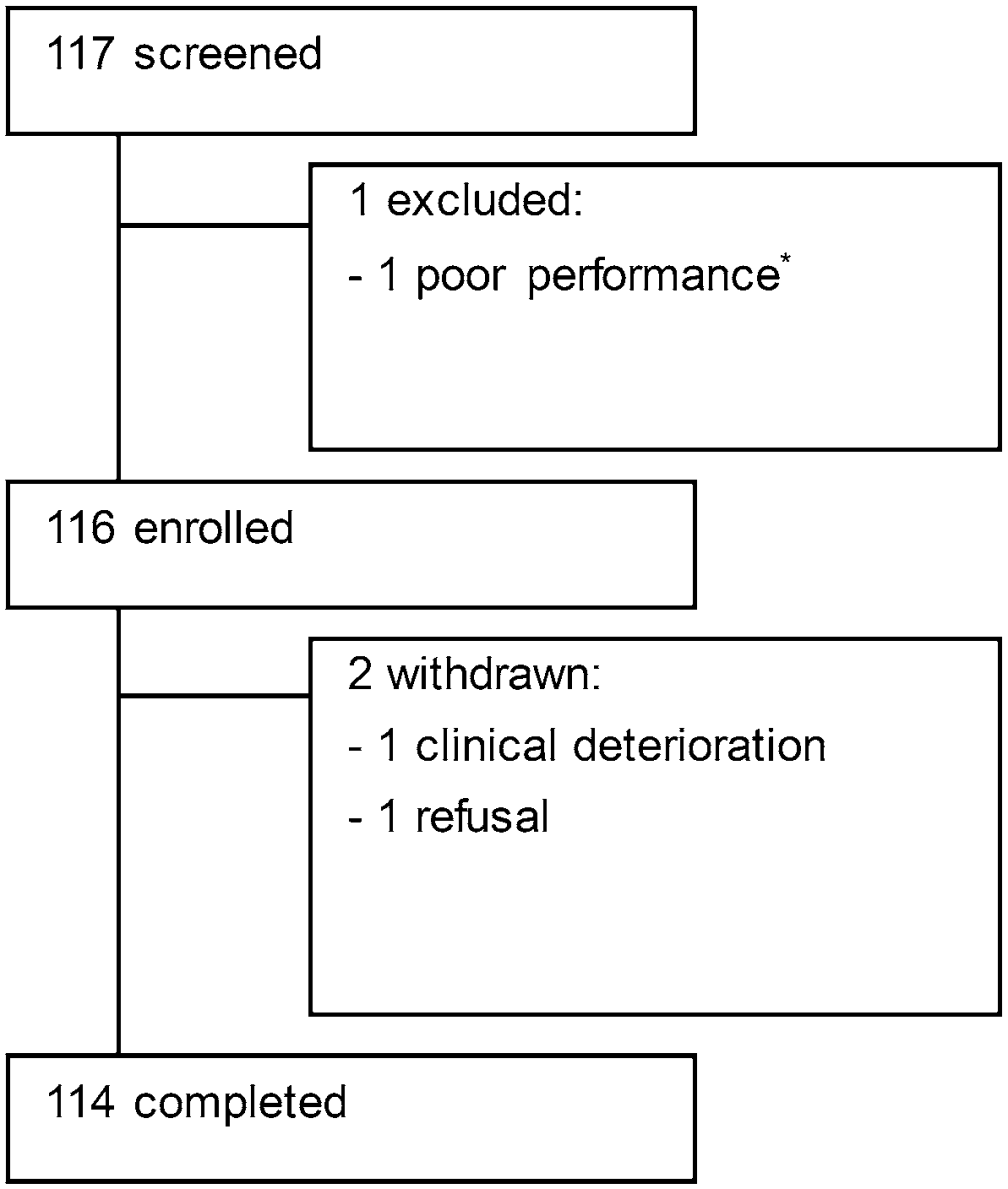
Study enrollment. Schematic overview of the screening, enrollment, and follow-up of the study participants. *Eastern Cooperative Oncology Group-Performance Status (ECOG-PS) > 2 or Karnofsky Performance Status (KPS) < 70.

From April to July 2021, a total of 117 consecutive patients underwent TSS for a pituitary tumor at the Samsung Medical Center in Seoul, Korea. Of a total of 117 patients, 116 were enrolled in the study, of whom 114 completed data collection and were included in the final analysis (Fig. 1). One patient was excluded due to postoperative hemorrhage, and 2 patients were discontinued due to a brain abscess and the patient's request for withdrawal. At baseline, the means of the Eastern Cooperative Oncology Group-Performance Status (ECOG-PS) and Karnofsky Performance Status (KPS) for the patients were 1 (median 1; range 1–2) and 80 (median 80; range 70–80), respectively. Table 1 presents the demographic data and clinical characteristics of patients who underwent TSS for a pituitary tumor. The mean age at the time of surgery was 52.4 years (range 21–80 years), and the patients included 57 men and 57 women. Among the patients, 91 (79.8%) worked before surgery. In terms of the histological type, PA was the most common in 94 patients (82.4%) followed by craniopharyngioma in nine patients (7.9%). According to the surgical approach, 89 patients (78.1%) received standard-TSS and 25 patients (21.9%) received extended-TSS and intraoperative CSF leak was observed in 49 patients (43.0%). Postoperative problems included CSF leak (1 patient, 0.9%), epistaxis (5 patients, 4.4%), hypopituitarism (14 patients, 12.3%), DI (14 patients, 12.3%), and syndrome of inappropriate secretion of antidiuretic hormone (3 patients, 2.6%).

**Table 1:** General and clinical characteristics of patients (N = 114).

Variables	n (%)/mean ± SD	Variables	n (%)/mean ± SD
Age (years)	52.4 ± 15.14	Preexisting TSS/craniotomy	10 (8.8)
Sex		Surgical approach I	
Male	57 (50.0)	Transseptal	91 (79.8)
Female	57 (50.0)	Endonasal	23 (20.2)
Smoking status		Surgical approach II	
Yes	11 (9.6)	Standard	89 (78.1)
No	103 (90.4)	Extended	25 (21.9)
Alcohol consumption		Intraoperative CSF leak	49 (43.0)
Yes	28 (24.6)	Postoperative problems	30 (26.3)
No	86 (75.4)	CSF leak	1 (0.9)
Occupation		Epistaxis	5 (4.4)
Yes	91(79.8)	Hypopituitarism, transient	14 (12.3)
No	20 (17.6)	Diabetes insipidus, transient	14 (12.3)
Unknown	3 (2.6)	SIADH	3 (2.6)
Histological type		Hemorrhage	3 (2.6)
PA, Non-functioning	81 (71.0)	Third cranial nerve palsy	2 (1.8)
PA, Growth hormone-producing	8 (7.0)	Additional treatment	
PA, Prolactin-producing	4 (3.5)	Lumbar drainage	4 (3.5)
PA, TSH-producing	1 (0.9)	Gamma knife radiosurgery	1 (0.9)
Craniopharyngioma	9 (7.9)	Steroid use	25 (21.9)
Meningioma	4 (3.5)	Hospital revisit	19 (16.7)
Chordoma	2 (1.8)	Postoperative hospital stay (days)	4.6 ± 2.33
Other^a^	5 (4.4)	Postoperative ICU stay	24 (21.1)

*CSF* Cerebrospinal fluid, *ICU* intensive care unit, *PA* pituitary adenoma, *SIADH* syndrome of inappropriate secretion of antidiuretic hormone, *TSS* transsphenoidal surgery, *TSH* thyroid stimulating hormone.

^a^Other: metastasis, arachnoid cyst, rathke cleft cyst, solitary fibrous tumor, and spindle cell oncocytoma.

### Time to return to ADL after surgery

**Figure 2:**
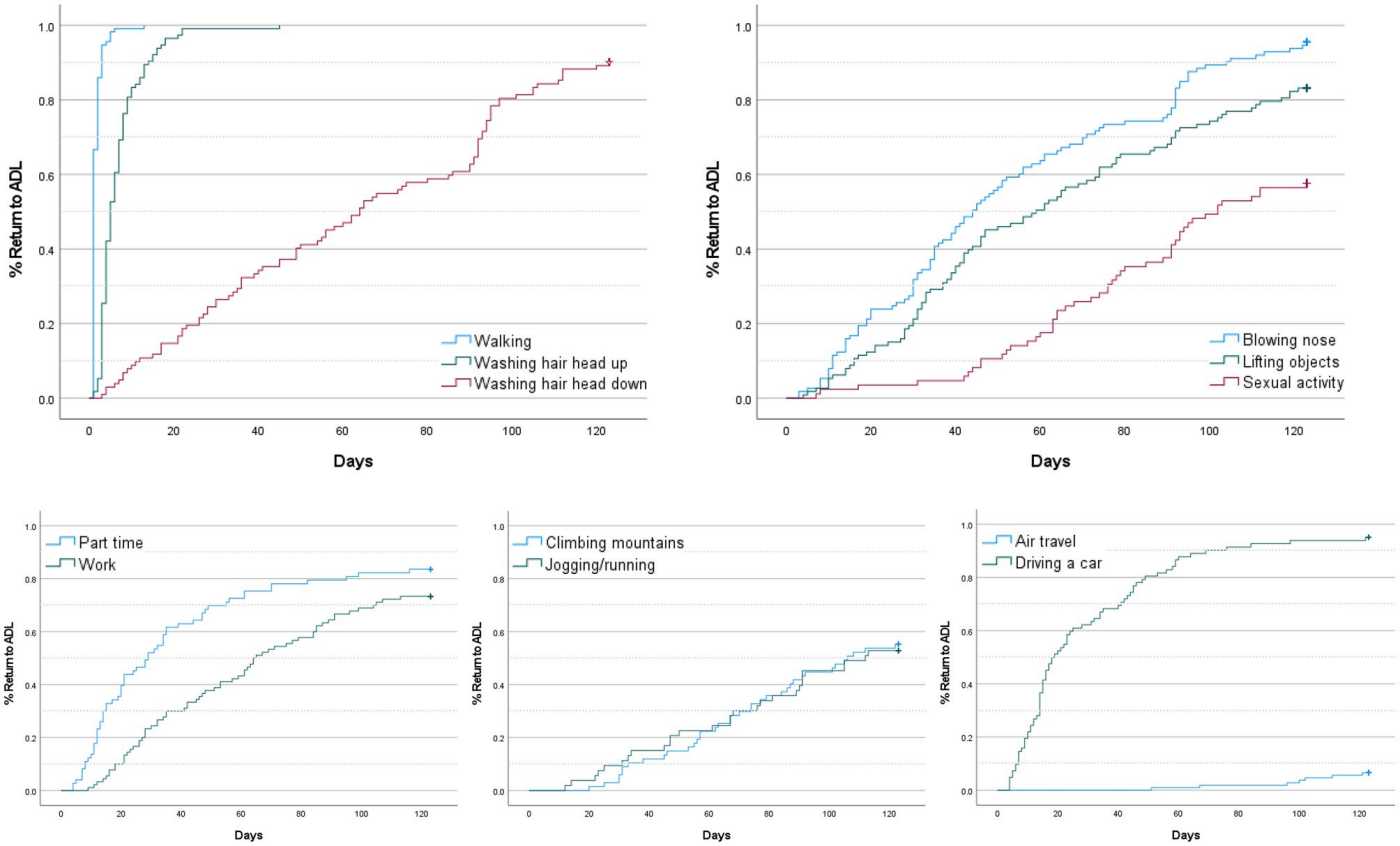
Percentage of return to ADL after surgery. The graphs illustrate the return rates according to the time elapsed after surgery for representative BADL (**A**) and IADL (**B**) elements.

In the elements of BADL, 50% of the patients took 5 days to wash their hair head up, approximately two months to wash their hair head down, approximately 1.5 months to blow their nose, approximately 2 months to lift/carry/move objects weighing more than 10 kg, and approximately 100 days to engage in sexual activity (Fig. 2, Table 2). Among the representative BADL elements (excluding lifting/carrying/moving objects, and sexual activity), more than 97% of the patients returned to performing them within 1 month (median: within 7 days). Lifting/carrying/moving objects > 10 kg, and sexual activity were not resumed by 16.8% and 42.4% of the patients, respectively, until 4 months after surgery (Table 2).

**Table 2:** Time to return to ADL after surgery (N = 114).

Activities		N/A (n, %)	Percentile of time to return (days)							Postoperative period (n, %)				
			0	10	25	50	75	90	100	1 m	2 m	3 m	4 m	4 m–
Basic ADL domains														
Mobility	Walking	0 (0.0)	1	1	1	1	2	3	13	114 (100.0)	–	–	–	–
Bathing/washing	Washing hair head up	0 (0.0)	1	3	3	5	8	14	45	113 (99.1)	1 (0.9)	–	–	–
	Washing hair head down	12 (10.5)	3	12	30	64	95	123	123^+^	27 (26.5)	23 (22.5)	21 (20.6)	21 (20.6)	10 (9.8)
	Taking a shower	0 (0.0)	1	3	3	5	8	15	23	114 (100.0)	–	–	–	–
	Blowing nose	1 (0.9)	3	11	26	44	89	103	123^+^	38 (33.6)	36 (31.9)	20 (17.7)	14 (12.4)	5 (4.4)
Dressing		0 (0.0)	1	1	1	2	3	5	22	114 (100.0)	–	–	–	–
Feeding/eating/drinking	Having a meal	0 (0.0)	1	1	1	1	2	3	7	114 (100.0)	–	–	–	–
	Coffee/tea	6 (5.3)	1	1	3	6	14	28	123^+^	97 (89.8)	7 (6.5)	1 (0.9)	2 (1.9)	1 (0.9)
Climbing stairs		0 (0.0)	1	3	4	7	12	20	45	110 (96.5)	4 (3.5)	–	–	–
Toilet use		0 (0.0)	1	1	1	1	2	3	16	114 (100.0)	–	–	–	–
Self-care in general		0 (0.0)	1	1	2	3	6	11	65	112 (98.2)	0 (0.0)	2 (1.8)	–	–
Grooming		0 (0.0)	1	2	3	5	7	15	26	114 (100.0)	–	–	–	–
Transferring (bed/chair/wheelchair/toilet/tub/shower/car)		0 (0.0)	1	1	1	2	3	7	24	114 (100.0)	–	–	–	–
Lifting/carrying/moving objects	Over 2 kg	0 (0.0)	2	4	8	14	23	32	92	102 (89.5)	8 (7.0)	4 (3.5)	–	–
	Over 10 kg	1 (0.9)	4	16	32	59	102	123^+^	123^+^	27 (23.9)	33 (29.2)	21 (18.6)	13 (11.5)	19 (16.8)
Sexual activity		29 (25.4)	7	48	68	102	123^+^	123^+^	123^+^	4 (4.7)	11 (12.9)	21 (24.7)	13 (15.3)	36 (42.4)
Instrumental ADL domains														
Work (also studying/volunteering/homemaking)	Part time	41 (36.0)	4	8	13	29	61	123^+^	123^+^	39 (53.4)	16 (21.9)	3 (4.1)	3 (4.1)	12 (16.5)
	Work (8 h/day)	24 (21.1)	9	21	32	65	123^+^	123^+^	123^+^	22 (24.4)	20 (22.2)	18 (20.0)	6 (6.7)	24 (26.7)
	Non-physical work	34 (29.8)	5	12	16	32	80	123^+^	123^+^	37 (46.2)	19 (23.7)	7 (8.7)	3 (3.7)	14 (17.5)
	Physical work	66 (57.9)	7	9	17	33	66	123^+^	123^+^	23 (47.9)	11 (22.9)	4 (8.3)	1 (2.1)	9 (18.8)
Housekeeping/chores		8 (7.0)	3	6	9	15	28	56	123^+^	84 (79.2)	15 (14.1)	4 (3.8)	1 (1.0)	2 (1.9)
Social activities		8 (7.0)	4	14	28	53	123^+^	123^+^	123^+^	32 (30.2)	32 (30.2)	11 (10.4)	4 (3.7)	27 (25.5)
Leisure time (hobbies, sports, vacation)	Hobbies (non-sports)	27 (23.7)	1	5	8	14	34	83	123^+^	64 (73.6)	13 (14.9)	6 (6.9)	0 (0.0)	4 (4.6)
	Wind instrument	111 (97.4)	80	123^+^	123^+^	123^+^	123^+^	123^+^	123^+^	0 (0.0)	0 (0.0)	1 (33.3)	0 (0.0)	2 (66.7)
	Climbing mountains	47 (41.2)	20	36	65	106	123^+^	123^+^	123^+^	6 (9.0)	10 (14.9)	14 (20.9)	7 (10.4)	30 (44.8)
	Jogging/running	61 (53.5)	12	31	67	112	123^+^	123^+^	123^+^	6 (11.3)	7 (13.2)	11 (20.8)	4 (7.5)	25 (47.2)
	Vacation	3 (2.6)	5	30	49	87	123^+^	123^+^	123^+^	12 (10.8)	30 (27.0)	17 (15.3)	15 (13.5)	37 (33.4)
Use of transport/travel around	Riding as a passenger in a vehicle/car	0 (0.0)	2	3	3	5	10	14	29	114 (100.0)	–	–	–	–
	Air travel	8 (7.0)	51	123^+^	123^+^	123^+^	123^+^	123^+^	123^+^	0 (0.0)	1 (0.9)	1 (0.9)	5 (4.7)	99 (93.4)
Communicating/expressing		0 (0.0)	0	1	1	1	2	6	24	114 (100.0)	–	–	–	–
Shopping (grocery, clothing, or other products)		0 (0.0)	1	2	3	8	14	23	64	108 (94.7)	5 (4.4)	1 (0.9)	–	–
Finances and administration (handling money, filling in forms)		4 (3.5)	1	3	4	9	16	25	123^+^	102 (92.7)	5 (4.6)	1 (0.9)	1 (0.9)	1 (0.9)
Driving a car		32 (28.1)	4	7	12	19	45	69	123^+^	52 (63.4)	20 (24.4)	4 (4.9)	2 (2.4)	4 (4.9)
Modern appliances ((mobile) phone, computer, laptop, tablet, Satnav)		5 (4.4)	0	1	1	1	3	4	123^+^	106 (97.3)	1 (0.9)	0 (0.0)	0 (0.0)	2 (1.8)
Managing own medication	Taking medicine	0 (0.0)	0	1	1	1	2	4	123^+^	113 (99.1)	0 (0.0)	0 (0.0)	0 (0.0)	1 (0.9)
	CAM therapy	5 (4.4)	1	6	22	49	106	123^+^	123^+^	39 (35.8)	27 (24.8)	9 (8.2)	10 (9.2)	24 (22.0)
	Using CPAP	113 (99.1)	17	17	17	17	17	17	17	1 (100.0)	–	–	–	–

123^+^, After 123 days; *ADL* Activities of daily living, *CAM* Complementary and alternative medicine, *CPAP* Continuous positive airway pressure, *N/A* Not applicable.

In the elements of IADL, 50% of the patients took approximately 1 month to begin part-time work, approximately 2 months to work 8 h a day, approximately 110 days to climb mountains and jog/run, and 19 days to drive a car. Only 6.6% of the patients resumed air travel within 4 months (Fig. 2, Table 2). Among the representative IADL elements, more than 79% of the patients resumed within 1 month, and more than 92% resumed within two months for elements excluding work, social activities, leisure, and driving a car (median values: within 15 days). Of the patients, 73.3% resumed working 8 h/day within 4 months. Regarding part time/work (8 h/day) and social activities, 16.5/26.7% and 25.5% of the patients, respectively, did not resume until 4 months after surgery. Also, when it comes to leisure and driving a car, 4.6–100.0% and 4.9% of the patients, respectively, did not resume until 4 months after surgery. Additionally, the median time for the activities included after 4 months for wind instrument, 17 days for using continuous positive airway pressure (CPAP), 6 days for drinking coffee/tee, and 49 days for complementary and alternative medicine (CAM) (Table 2).

### Difference in the time to return to ADL according to clinical characteristics

Upon looking at the difference in the time to return to ADL after surgery according to the clinical characteristics of patients who underwent TSS for a pituitary tumor (Table 3), the patients who received extended-TSS were all slower than those who received standard-TSS for the activities mentioned below: walking (p = 0.021), washing hair head up (p < 0.001), taking a shower (p = 0.003), dressing (p = 0.014), climbing stairs (p = 0.001), toilet use (p = 0.033), self-care in general (p = 0.048), grooming (p = 0.013), transferring (p = 0.005), finances and administration (p = 0.029), using modern appliances (p = 0.007), and managing own medication (p = 0.009). Patients with postoperative problems were slower than those without them, for walking (p = 0.002), washing hair head up (p = 0.001), taking a shower (p = 0.001), dressing (p = 0.004), having a meal (p = 0.002), climbing stairs (p = 0.035), toilet use (p = 0.004), transferring (p < 0.001), communicating/expressing (p = 0.004), using modern appliances (p = 0.003), and managing own medication (p = 0.038), while were faster for climbing mountains (p = 0.032).

**Table 3:** Differences in the time to return to ADL according to the clinical characteristics (including significant variables).

Variables		Median (Q1, Q3) or mean ± SD (days)/r	p	Variables		Median (Q1, Q3) or mean ± SD (days)/r	p
Basic ADL domains							
Walking				Age		0.236	0.011
Surgical approach II	Standard	1.0 (1.0,2.0)	0.021	Occupation	Yes	7.0 (4.0,12.0)	0.039
	Extended	2.0 (1.0,2.0)			No	8.0 (5.5,19.5)	
Postoperative problems	Yes	2.0 (1.0,3.0)	0.002	Histological type	PA/RCC/AC	7.0 (4.0,12.0)	0.009
	No	1.0 (1.0,2.0)			Other	12.0 (6.0,17.0)	
Lumbar drainage	Yes	2.5 (1.5,8.0)	0.036	Surgical approach II	Standard	6.0 (4.0,11.0)	0.001
	No	1.0 (1.0,2.0)			Extended	12.0 (7.0,17.0)	
Steroid use	Yes	2.0 (1.0,2.0)	0.002	Postoperative problems	Yes	12.0 (6.0,17.0)	0.035
	No	1.0 (1.0,2.0)			No	7.0 (4.0,11.5)	
Washing hair head up				Toilet use			
Histological type	PA/RCC/AC	5.0 (3.0,8.0)	0.002	Surgical approach II	Standard	1.0 (1.0,2.0)	0.033
	Other	8.5 (6.0,13.0)			Extended	2.0 (1.0,3.0)	
Surgical approach II	Standard	4.0 (3.0,7.0)	< 0.001	Postoperative problems	Yes	2.0 (1.0,4.0)	0.004
	Extended	8.0 (6.0,13.0)			No	1.0 (1.0,2.0)	
Postoperative problems	Yes	8.0 (5.0,13.0)	0.001	Steroid use	Yes	2.0 (1.0,3.0)	0.033
	No	4.5 (3.0,7.0)			No	1.0 (1.0,2.0)	
Lumbar drainage	Yes	15.0 (13.5,17.0)	0.003	Self-care in general			
	No	5.0 (3.0,8.0)		Surgical approach I	Transseptal	3.0 (2.0,7.0)	0.034
Steroid use	Yes	8.0 (5.0,13.0)	0.002		Endonasal	2.0 (1.0,3.0)	
	No	5.0 (3.0,7.0)		Surgical approach II	Standard	3.0 (1.0,5.0)	0.048
Washing hair head down					Extended	30.0 (20.0,90.0)	
Age		− 0.210	0.044	Intraoperative CSF leak	Yes	3.0 (2.0,9.0)	0.016
Preexisting TSS/craniotomy	Yes	21.0 (8.0,45.0)	0.005		No	3.0 (1.0,4.0)	
	No	62.0 (31.5,92.5)		Grooming			
Taking a shower				Sex	Male	40.0 (20.0,50.0)	0.001
Age		0.185	0.049		Female	6.0 (4.0,10.0)	
Histological type	PA/RCC/AC	4.0 (3.0,7.0)	< 00.001	Histological type	PA/RCC/AC	4.0 (3.0,7.0)	0.016
	Other	11.0 (6.0,13.0)			Other	5.5 (4.0,15.0)	
Surgical approach II	Standard	4.0 (3.0,7.0)	0.003	Surgical approach II	Standard	4.0 (3.0,7.0)	0.013
	Extended	8.0 (5.0,13.0)			Extended	5.0 (4.0,15.0)	
Postoperative problems	Yes	8.0 (5.0,13.0)	0.001	Steroid use	Yes	7.0 (4.0,15.0)	0.003
	No	4.0 (3.0,7.0)			No	4.0 (3.0,7.0)	
Lumbar drainage	Yes	13.5 (12.0,15.0)	0.008	Transferring (bed/chair/wheelchair/toilet/tub/shower/car)			
	No	5.0 (3.0,8.0)		Age		0.273	0.003
Steroid use	Yes	7.0 (5.0,13.0)	0.004	Occupation	Yes	1.0 (1.0,3.0)	0.012
	No	4.0 (3.0,7.0)			No	2.0 (1.0,8.0)	
Blowing nose				Surgical approach II	Standard	10.0 (10.0,30.0)	0.005
Preexisting TSS/craniotomy	Yes	24.5 (14.0,35.0)	0.042		Extended	3.0 (1.0,6.0)	
	No	44.5 (28.0,75.0)		Intraoperative CSF leak	Yes	2.0 (1.0,5.0)	0.010
Surgical approach I	Transseptal	47.5 (30.0,90.0)	0.005		No	1.0 (1.0,2.0)	
	Endonasal	30.0 (17.0,40.0)		Postoperative problems	Yes	3.0 (2.0,6.0)	< 0.001
Dressing					No	10.0 (10.0,30.0)	
Surgical approach II	Standard	1.0 (1.0,2.0)	0.014	Lumbar drainage	Yes	12.5 (5.5,17.5)	0.006
	Extended	2.0 (1.0,4.0)			No	1.0 (1.0,3.0)	
Postoperative problems	Yes	2.0 (1.0,5.0)	0.004	Steroid use	Yes	2.0 (2.0,5.0)	0.008
	No	1.0 (1.0,2.0)			No	1.0 (1.0,3.0)	
Having a meal				Lifting/carrying/moving objects over 2 kg			
Postoperative problems	Yes	2.0 (1.0,3.0)	0.002	Sex	Male	10.0 (5.0,19.0)	0.009
	No	1.0 (1.0,2.0)			Female	16.0 (10.0,25.0)	
Steroid use	Yes	2.0 (1.0,3.0)	0.046	Sexual activity			
	No	1.0 (1.0,2.0)		Intraoperative CSF leak	Yes	84.35 ± 18.00	0.022
Climbing stairs					No	660.19 ± 280.72	
Instrumental ADL domains							
Work (8 h/day)				Histological type	PA/RCC/AC	50.0 (30.0,100.5)	0.049
Preexisting TSS/craniotomy	Yes	21.0 (15.0,23.5)	0.027		Other	7.0 (5.0,10.0)	
	No	51.0 (28.0,76.0)		Communicating/expressing			
Non-physical work				Occupation	Yes	10.0 (10.0,20.0)	0.024
Sex	Male	18.0 (12.5,30.0)	0.010		No	1.5 (1.0,4.0)	
	Female	34.0 (20.5,61.5)		Postoperative problems	Yes	2.0 (1.0,4.0)	0.004
Housekeeping/chores					No	10.0 (10.0,20.0)	
Age		− 0.229	0.019	Finances and administration			
Climbing mountains				Surgical approach II	Standard	80.0 (40.0,150.0)	0.029
Sex	Male	80.05 ± 26.52	0.002		Extended	14.0 (7.5,29.5)	
	Female	52.69 ± 21.80		Driving a car			
Postoperative problems	Yes	48.14 ± 20.98	0.032	Sex	Male	14.5 (9.0,32.0)	0.009
	No	72.90 ± 27.46			Female	27.5 (16.0,51.0)	
Riding as a passenger in a vehicle/car				Preexisting TSS/craniotomy	Yes	120.0 (40.0,180.0)	0.049
					No	19.0 (12.0,43.5)	

*AC* Arachnoid cyst, *ADL* Activities of daily living, *CSF* Cerebrospinal fluid, *PA* Pituitary adenoma, *RCC* Rathke cleft cyst, *TSS* Transsphenoidal surgery.

The time taken to resume the following activities (among BADL) is described as follows: older patients at the time of surgery (p = 0.044) and patients who had a previous TSA/craniotomy (p = 0.005) resumed “washing their hair head-down” earlier than younger patients and those who did not. Patients who had a previous TSA/craniotomy resumed “blowing their nose” earlier than those who did not (p = 0.042), whereas patients who underwent a transseptal approach took longer than those who underwent an endonasal approach (p = 0.005). “Lifting/carrying/moving objects” was resumed sooner in men than in women (p = 0.009), and sexual activity took longer in patients who had an intraoperative CSF leak than in patients who did not (p = 0.022). The time taken to resume the following activities (among IADL) is described as follows: patients who had a previous TSA/craniotomy resumed work (8 h/day) earlier than those who did not (p = 0.027), and men resumed non-physical work earlier than women (p = 0.010). Men took longer to “climb mountains” than women (p = 0.002), whereas patients with postoperative problems resumed earlier than those without (p = 0.032). “Driving a car” was resumed sooner in men than in women (p = 0.009) and in patients who had a previous TSA/craniotomy than in those who did not (p = 0.049).

## Discussion

This study attempted to present the basic data to provide information and guidelines on the “appropriate return period,” to the medical staff and patients by examining the time to return to ADL after TSS for a pituitary tumor. With the exception of lifting/carrying/moving objects and sexual activity, more than 97% of patients were observed to resume BADL within one month for most elements whereas for most elements of IADL, it took up to two months for more than 92% of the patients, with the exception of work, social activities, leisure, and driving a car. The results from another study showed that the disease burden, mental and physical functioning, and visual functioning improved within 6 weeks after pituitary tumor surgery^6^. Additionally, the guidelines recommended by the German pituitary working group reported that all elements of daily activity, sports, and occupation can be implemented within 3 months after surgery^5^; however, patients included in this study had a late return to ADL. More patients than expected independently decided when to return to the activities. Based on the results of this study, it is possible to prevent the patients from returning too early for elements that may be risky. Conversely, the patients who delay their return may be encouraged to return faster on the basis that no special problems have occurred in patients who returned earlier. Moreover, in this study, it can be observed that there was a difference in the time to return after surgery depending on factors, such as the extended- or standard-TSS and the presence or absence of postoperative problems. In particular, it should be considered that the return time is delayed in the case of extended-TSS compared to the case of standard-TSS.

The time to return to the individual elements reported in this study was compared with the recommended time suggested by the German pituitary working group. The working group suggested that intraoperative CSF leak, intracranial air, hyponatremia, and brain surface involvement by tumor or resection should be checked, adding that the recommendations indicate the minimum time interval after surgery^5^. In this study, the median value for “washing hair head up” was 5 days, and it was related to the histological type of the tumor, standard/extended-TSS, postoperative problems, and lumbar drainage or steroid usage after surgery. It is similar to the recommended “hair washing time” of less than 1 week. Of the work elements, a crucial patient-centered outcome parameter, the median value of time to return to “8-h work per day” was approximately two months, and difference was present depending on the past surgical history. The recommended time was 2–3 weeks for non-physical work and 4–6 weeks for physical work. The patients in this study returned to the median value of approximately 1 month for both non-physical and physical work, slightly later in the former and slightly earlier in the latter compared to the recommended time. The time to return to non-physical work was related to the sex. According to the results, 86% of patients who had a job before surgery remained employed after 6 months^6^ and there was no effect on the long-term follow-up compared to the general population for returning to work^9^. It might be necessary to investigate by extending the follow-up period. In the case of “mountain climbing,” which applied to the most patients in the sports elements, it was performed at the median value of 106 days, and sex and postoperative problems were related factors. Compared to the 3–12 week recommendation for all sports, the return was delayed. In the case of driving, it was implemented at the median value of 19 days, which is also slightly delayed compared to the 1–2 week recommendation. In this study, it differed according to the sex and past surgical history of the individual.

In addition, the timing of washing hair head down (median value of 64 days), blowing nose (44 days vs. 3–4 weeks), lifting heavy objects (59 days vs. 4–6 weeks), sexual activity (102 days vs. 1–2 weeks), wind instrument (after 4 months vs. 6 weeks), and using CPAP (17 days vs. 3–4 weeks) were different from the recommended guidelines. Therefore, it is necessary to establish a new guideline by reviewing and negotiating the surgeon’s opinions and patient’s perspective from various angles. This will provide useful information about the possible return period for ADL, including elements that patients are actually curious about in the clinical field, such as drinking coffee/tea, air travel, and CAM therapy.

This study had the following limitations. First, although the performance status did not clearly deteriorate below the criterion during the study period, the time to return may have been somewhat delayed, as we included some patients who were in various conditions and showed slow recovery and temporary deterioration of their status. Second, it should be taken into account that there was a difference in the understanding of the elements depending on the patient. Third, it may have been measured conservatively because the patients were asked to write down the time when it was executed rather than the time when it was judged that it is possible, and there was a tendency for patients to hesitate or to delay by caregivers. Fourth, the responses may be limited due to the effect of time limit, seasonal factors, and the COVID-19 situation. In particular, there is a possibility that it may not have been completed for elements, such as sports or air travel. This should be taken into account when interpreting the results, and it is suggested to extend the postoperative follow-up period in the future. Improvements are needed in research methods and processes, such as reorganizing the tool and computerizing the survey methods in consideration of extending the follow-up period. Nevertheless, this research activity is an initial effort to understand the recovery process after TSS in pituitary tumor patients, and the results of this study can be used as the basic data to provide daily life management guidelines for pituitary tumor patients after TSS.

## Conclusions

The time to return with respect to various elements of ADL was measured after pituitary tumor surgery. Additionally, the time to return was different for each patient and varied according to several factors. Based on these results, it will be possible to provide practical information and guidelines on the time to return to ADL after TSS in pituitary tumor patients. Moreover, it will maintain the function and well-being of daily life after surgery by reducing the post-surgical complications, uncertainty about recovery and daily life, and helping patients return to their daily life in a timely manner.

## Methods

We conducted a prospective study to assess the timing of return to ADL after TSS for a pituitary tumor. All experimental protocols were approved by the Institutional Review Board of the Samsung Medical Center (Study number: 2021-03-113), and written informed consent was obtained from all the patients. All methods were performed in accordance with the relevant guidelines and regulations. The inclusion criteria were as follows: (a) Age ≥ 18 years; (b) Histologically confirmed tumor; (c) Patients who underwent tumor removal by TSS; (d) ECOG-PS ≤ 2 or KPS ≥ 70 at discharge after surgery; (e) Patients with no communication problems, who understood the purpose of the study, and consented to participate; (f) Performance status did not worsen below the criterion during the study period; (g) No re-TSS or craniotomy was performed for tumor progression/recurrence or residual tumors during the study period. Patient education on daily life was carried out before discharge. As per our established protocol, we recommended that patients should avoid certain activities such as work with heavy physical activity, hair washing with head down, air plane boarding, blowing the nose and sneezing for a duration of 1 or 3 months following TSS. At an outpatient visit, the patient was allowed to resume these activities as deemed appropriate by their outpatient physician. The outpatient physician made a comprehensive assessment of the presence or absence of postoperative complications, surgical approach, postoperative blood sampling results, etc., and indicated to each patient when he or she could return to each ADL.

Electronic medical records were reviewed to collect general and clinical characteristics of the patients. A self-recording sheet was developed to measure when patients returned to ADL after surgery, minimizing the recall period. The ADL elements in the self-recording sheet were constructed reflecting the clustered list of basic and instrumental activities by Oort et al.^10^ and the detailed list of activities provided on the websites of 27 hospitals or institutions. The ADL were broadly classified into 15 elements of “basic” ADL (BADL) and 31 elements of “instrumental” ADL (IADL), with each element presented in order of the measurement frequency. It was divided into sub-activities for four BADL elements and four IADL elements, with 35 elements and 50 elements, respectively, for a total of 85 elements. BADL included activities that enable basic survival and well-being, such as bathing, dressing, eating, and using the toilet, while IADL included more complex activities than BADL, such as housekeeping, grocery shopping, managing finances, phone calls, and taking medication. The content validity of the developed self-recording sheet was evaluated by a group of five experts: one neurosurgeon, two head nurses in the neuro ward, one coordinator, and one physician assistant. As a result, the scale-level content validity index using the universal agreement method (S-CVI/UA) was 0.78, and the scale-level content validity index using the averaging method (S-CVI/Ave) was 0.94. The item-level content validity index (I-CVI) ranged from 0.60 to 1.00.

It was designed to input the return time for each ADL element. The “time to return to ADL” referred to the time elapsed after surgery until the point in time when resumption of preoperative ADL became possible postoperatively. If the activities had not been performed before surgery and there was no plan to perform them in the future, they were marked as “not applicable.” In this study, we collected self-reported sheets that documented the patients' ADL return times from April to November 2021. At discharge and at 1, 2, 3, and 4 months after surgery, it was checked whether the patient had written the execution time for each ADL element, and the missing parts were encouraged to be filled in. A copy of the sheet was kept at discharge and one month after surgery (at the time of visit) to reprovide in case of loss, and the final sheet was retrieved four months after surgery. When a hospital visit was scheduled, the patient was contacted in advance to remind them to fill and bring the self-recording sheet.

### Statistical analysis

A statistical analysis was performed with SPSS version 25.0 (IBM Corporation, Armonk, New York, USA). The general and clinical characteristics were expressed as numbers and percentages or mean and standard deviation. A Kaplan–Meier survival analysis and percentile of time to return were performed to assess the time to return to ADL. In addition, the number and percentage of each period were obtained to evaluate the ADL return rate by period after surgery. For the difference in the time to return to ADL according to the general and clinical characteristics, the Mann–Whitney U test, Kruskal–Wallis test, independent samples t-test, ANOVA, and Spearman correlation were used after a normality test using the Kolmogorov–Smirnov and Shapiro–Wilk tests. The statistical analyses were guided by a statistician.

## Data Availability

The datasets generated and analysed during the current study are available from the corresponding author on reasonable request.
